# P216: Blood culture contamination as an indicator of hand hygiene compliance

**DOI:** 10.1186/2047-2994-2-S1-P216

**Published:** 2013-06-20

**Authors:** M-N Chraiti, W Zingg, A Gayet-Ageron, D Pittet

**Affiliations:** 1 Infection Control Programme, University of Geneva Hospitals, Geneva, Switzerland

## Introduction

Hand hygiene (HH) monitoring by direct observation is the gold standard for compliance measure but is highly resource consuming. Indirect indicators could help to overcome resource constraints that are identified as a major obstacle to evaluate and feedback on HH practices. HH compliance may impact on blood culture contamination (BCC) rate.

## Objectives

The objective of the study was to explore the association between HH compliance and BCC in a university affiliated tertiary care hospital.

## Methods

Ecological study combining prospective laboratory-based surveillance of bacteraemia and repeated direct HH observation over a study period of 15 consecutive quarters between 2009 and 2012. Each positive blood culture result was checked for aetiology using patient chart and laboratory data. HH compliance observations were conducted among all healthcare workers based on the World Health Organization HH guidelines. Probable BCC and nurse HH compliance were included in the analysis. Sub analysis included data from the intensive care unit (ICU) and the emergency room (ER).

## Results

In total, 5388 positive blood culture episodes were identified, of which 677 (12.6%) were BCC, mainly due to skin contaminants (91%). 154 (22.7%) and 217 (32.1%) were attributed to ICU and ER, respectively. A significant trend towards less BCC was found in the ICU (IRR [95%CI]: 0.96 [0.93-0.99]; P=0.047). A total of 13,393 HH opportunities were observed in the hospital, 1882 in the ICU, and 379 in the ER. The total numbers of HH opportunities before an aseptic task for hospital, ICU, and ER were 2558, 352, and 62, respectively. Average (range) HH compliance for hospital, ICU, and ER was 0.69 (0.59-0.76; IRR: 1.01; P<0.001), 0.62 (0.53-0.70; P=0.86), and 0.50 (0.22-0.74; P=98), respectively. A significant association between HH and BCC was found in the ICU (IRR [95%CI]: 0.99 [0.98-0.99]; P=0.004). No other association was identified.

## Conclusion

Although an association for overall HH compliance and BCC was shown, this was not confirmed for the indication “before an aseptic task”, neither in the ER due to a lack of power. This finding needs to be verified with larger samples per time unit before concluding whether useful or not.

## Disclosure of interest

None declared

